# Onset Crime Typology of Sexual Offenders and Their Differences on Specialization and Risk Factors

**DOI:** 10.3389/fpsyg.2022.845670

**Published:** 2022-05-25

**Authors:** Chien Huang, Sheng-Ang Shen, Tao-Hsin Tung

**Affiliations:** ^1^Department of Clinical Psychology, College of Medicine, Fu-Jen Catholic University, New Taipei City, Taiwan; ^2^Department of Crime Prevention and Correction, Central Police University, Taoyuan, Taiwan; ^3^Evidence-Based Medicine Center, Taizhou Hospital of Zhejiang Province Affiliated With Wenzhou Medical University, Linhai, China

**Keywords:** sexual offenders, onset typology, sex crime, specialization, risk factors

## Abstract

In those theories or empirical-evident model of sexual offending, they all recognized which major life event would cause the sex offense in some conditions, therefore the onset crime of sexual offenders were not only a mark of personal history, but also could reflect the heterogeneity of sexual offenders. Our purpose is to study the onset crime typology of sexual offender and their difference in specialization, problem of psychology marks, and negative developmental experiences. We analyzed the pre-conviction data from 3,750 sexual offenders and their risk assessment data. The research results found that onset typology of sex crime would persist their criminal career into sexual offending, and through the group comparisons, the study pointed out differences in risk factors domain and adverse development experiences. We also discussed those research results and their meaning of risk management.

## Introduction

The severe and long-term physical and mental health issues caused by sexual assaults should be a major public concern (Hutchings and Dutton, 1993; McMahon, 2000; Cloutier et al., 2002; Elliott et al., 2004). Many of the existing studies on sexual offending have focused on the risk factors and the resulting psychopathological or etiological models, such as the etiological model of sexual recidivism risk proposed by Ward and Beech (2006) or the descriptive models developed by Ward and other research teams based on interview data and the grounded theory (Ward and Hudson, 1998, 2000; Hudson et al., 1999; Polaschek et al., 2001). These models focus on understanding the evolution process of sexual offending, which starts from the distal factors or background factors rather than from the sexual offenders themselves. Studies have also attempted to analyze the responses of sexual offenders to sexual arousal-related stimuli to infer the underlying cognitive structures and operation process characteristics. The causes of sexual assaults exhibit three primary components: certain key events in development, the shaping of risk factors, and sexual offenses derived from internal and external stimuli. Therefore, Thornton (2002), and Beech et al. (2002) all highlighted that sexual offenders not only exhibit differences in their static risk factors, but their dynamic risk factors also vary. When sexual offenders focus on dynamic risk factors on different levels, it results in different types of offending pathway described by Ward and Siegert (2002).

Both descriptive and etiological models of sexual offending and the characteristics of cognitive structures and operations indicate that aspects of previous experiences in the lives of sexual offenders have an impact on the trajectory and shaping of sexual assault, which cannot be ignored, particularly aspects that open them up to violence or offenses. For instance, Malamuth (2003) adopted an empirical approach and used a confluence model constructed based on the tests and follow-ups of general adults in communities, a path that was named the impersonal sex path, to emphasize that the sexual aggression of sex offenders is propelled by a series of abusive experiences and early criminal or deviant behavior in their upbringing. However, past studies have rarely discussed the snowball effect in the trajectories of the criminal career of sexual offenders (Lussier and Brassard, 2015; Smallbone and Cale, 2015).

Using the criminal records of over 3,000 sexual offenders, Huang et al. (2017) study employed group-based trajectory modeling to analyze the variations in the criminal career trajectories of four categories of sexual offenders. They further inferred that the age of onset for sexual offending has a crucial impact on sexual and general recidivism, and this view is consistent with the integrated theory of sexual offending proposed by Ward and Beech (2006), in which the onset of sexual offending is a significant event in an individual’s life and changes the individual’s ecological environment and learning experience. Both Thakker and Ward (2015) indicated that onset is one of the key factors influencing the changes in the criminal career of a sexual offender. Some of researches already found that offending types at a stark point of the life course has certain meanings in different type of offenders (e.g., Mazerolle et al., 2010; DeLisi et al., 2015). Regardless those theoretical perspectives, we still need to have a close investigation on whether the initial offense categories shape the criminal career as specialization or versatility offenders that are largely under researched in sexual offender in Taiwan.

Past studies on sexual offending have mostly focused on the fact that a significant correlation exists between the age at which sexual violence is committed and the subsequent sexual recidivism of the sexual offender (Hirschi and Gottfredson, 1983; Barbaree and Blanchard, 2008). However, Lussier and Brassard (2015) noted that the age of onset of sexual offending is important because it marks the situational context and environmental factors that initiate sexual offending. The other study examined the recidivism risk of sexual offenders based on their criminal records and indicated that past investigations of the rates of sexual recidivism overlooked the versatility of crime. They found that for those whose first official criminal record was a sexual offense, the risk of them committing a sexual offense again within 5 years was 13%; however, for those whose first official criminal record was a non-sexual crime, the risk of them committing a sexual offense within 5 years was over 40%. The results above demonstrate the possibility of variations in a sexual offender’s criminal career. If it begins with sexual offending, the risk or possibility that it will be oriented toward sexual crime is close to the risk or possibility that it will be oriented toward non-sexual crime (13 vs. 15%). However, if a criminal career begins with a non-sexual crime, there is a greater chance that the offender will commit a sexual crime in the future (41 vs. 29%). No matter how these are interpreted though, it seems that criminal careers that begin with a non-sexual crime are likely to be versatile. With regard to the integrated theory of sexual offending, Ward and Beech (2006), Smallbone and Cale (2015), or other theoretical perspectives of sexual offending mentioned that previous criminal outcomes will influence an offender’s next offense. However, these researchers did not discuss whether recidivism ultimately leads toward specialized or versatile criminal patterns. Smallbone and Wortley (2004) also mentioned that before criminals first sexually offend, they are likely to have already committed non-sexual offenses, such as violent offenses, property offenses, or addiction. The research results above lead us to the following question worth exploring further: does the onset typology of sexual offenders also reflect essential differences in their sexual offending?

Thus, the objective of this study was to group sexual offenders by their onset typologies and compare their criminal characteristics. Because there is no consensus used crime categories, our study tried to referenced the research strategies from different studies to choose this four type of onset crime, and it was also convention in criminology (e.g., Mazerolle et al., 2000; Harris et al., 2009; Harris, 2012). We referred to the analysis method used by Harris (2012), who advocates that onset typology exerts a critical impact on subsequent criminal career. We utilized information from official criminal records (criminal record files) and focused on four onset typologies: sexual offenses, violent offenses, property offenses, and addiction. We conducted in-depth analyses to compare the specialization, risk factor levels, and basic criminal variables of the onset typologies in order to understand the nature of the careers of sexual criminals.

## Materials and Methods

### Research Procedure

Under the legal procedure of Taiwan, all sexual offenders should get into community treatment in order to monitoring and make sure public safety. Therefore, first step of community treatment is to do risk assessment, all information is imported into a special database system, which is called “National Domestic Violence, Sexual Assault, and Children-Juvenile Protection Information System,” it belongs to Ministry of Health and Welfare of Taiwan. This study was a research project commissioned by same unit (project case no. M04B3252). All risk assessment done by trained therapist.

Research data were delinked export from this database system, and then, in accordance with the regulations of the Personal Data Protection Act, a case handler anonymized all of the required data before providing the data over to our research team, such that the file for each sample was only marked with a case number. The procedures and methods employed in this study were reviewed and approved by the Institutional Review Board of Fu Jen Catholic University (project approval no. C103152).

In order to analysis onset group typology, our research team looked for the Offense Determination Table and transcribed the criminal record data by age at the time of offense and by the type of offense. Then we also extracted the Static-99, acute dynamic risk factors, stable dynamic risk factors, and the four items of developmental history (any childhood sexual victimization, poor family relationship, maladaptive in school, and any conduct problems under 18) from the assessment data as group comparison variables and used them to retrospective the potential influence of the onset offense typology.

### Research Participants

All obtained data regarding 8,370 sexual offenders from 2007 to June of 2015 from the database system. Inspecting the completeness of the data and removing any samples lacking criminal record data for onset typology analysis resulted in data from 3,750 sexual offenders. Furthermore, we removed samples with overly incomplete data, that is when lack of any one of index sexual offense information, criminal records, and risk scales, research team would define them as overly incomplete data and excluded for data analysis. Followed this procedure, which resulted in complete risk assessment data from 819 sexual offenders for group comparison analysis. As shown in Table 1, the mean age of onset typology group was 38 years old (standard deviation: 13.47 years). On average, the official criminal records could be traced back 8.68 years in the past. The mean age of the samples in the group difference comparison was close to the previous mean age. Further examination of the age and how far back the data could be traced revealed that the differences between two groups with regard to these two variables were not significant. In addition, we observed whether the research samples had any prior convictions aside from the most recent sexual assault that was recorded in the information system. Our data analysis revealed that approximately 55–60% of the samples in two groups had prior criminal records of any type, and 82–85% had two or more prior sexual assaults. The chi-square tests for these two variables indicated no significant differences, showing that the results of the group difference comparison research can provide reference.

**TABLE 1:** Demographic characteristics of the samples.

	Data pool of onset group (*n* = 3,750)	Data pool for group comparison (*n* = 819)
Age (SD)	37.91 (13.47)	38.89 (13.72)
average years of follow-up^1^	8.68	8.98
	*n* (%)	*n* (%)
**Any pre-conviction**		
Yes	1661 (44.29)	327 (39.93)
No	2089 (55.71)	492 (60.07)
Sexual offense ≥ 2		
Yes	548 (14.61)	146 (17.83)
no	3202 (85.39)	673 (82.17)

*The official criminal records could be traced back to first offense.*

### Research Instruments

#### Criminal Record Information

The official criminal record data provided by the Offense Determination Table (i.e., criminal record files) of the “National Domestic Violence, Sexual Assault, and Children-Juvenile Protection Information System” included length of sentences, the criminal charge, and the personal criminal history of the sexual offenders. We focused on four types of offenses: sexual offenses, violent offenses, property offenses, and addiction, as categorized by Lussier et al. (2010). We therefore transcribed the criminal record data by age at the time of offense and by the type of offense. All of our research participants came from a special database system just for sexual offenders in Taiwan, so we did select their pre-convictions to find out four types of onset. In our research data, number and proportion of onset typology are: sex (*n* = 488, 59.6%), violence (*n* = 78, 9.5%), property (*n* = 121, 14.8%), and addiction (*n* = 37, 4.5%). We listed in Table 2.

**TABLE 2:** One-way ANOVA of different onset typology groups.

Variables	Sex	Violence	Property	Addiction	*F* value
Number of offender (%)	488 (59.6)	78 (9.5)	121 (14.8)	37 (4.5)	
Number of sexual offenses	1.39 (0.81)	1.04 (0.69)	1.31 (0.87)	0.89 (0.70)	4.92***
Age of first offense	31.65 (13.65)	26.90 (9.55)	26.63 (8.65)	25.8 (6.96)	6.24***
Intervals of any offense (years)	3.12 (2.51)	3.05 (1.42)	3.02 (1.47)	2.86 (1.42)	9.87***
Stable dynamic risk factor scores	4.21 (2.19)	4.72 (2.40)	4.99 (2.00)	4.22 (2.32)	3.19**
Acute dynamic risk factor scores	2.69 (1.82)	2.97 (2.14)	3.39 (1.86)	2.73 (1.85)	3.34**
Static-99	2.51 (1.30)	3.05 (1.42)	3.02 (1.47)	2.86 (1.42)	4.98***
Victim types					
Adult female	125 (25.6)	32 (41.0)	47 (38.8)	19 (51.4)	
Intra-family child	23 (4.7)	3 (3.8)	6 (5.0)	1 (2.7)	
Extra-family child	16 (3.3)	3 (3.8)	2 (1.7)	2 (5.4)	
Adolescence^1^	260 (53.3)	30 (38.5)	51 (42.1)	11 (29.7)	
Gang rape	5 (1.0)	2 (2.6)	0	0	
others	1 (0.2)	0	1 (0.8)	0	

*^1^Adolescence means victim age between age 12 to 18.*

**p < 0.01, ***p < 0.001.

However, our research found significant differences in onset group comparison of number of sexual offenses, age of first offense, and intervals of any offense. For further analysis, research results pointed out the sex onset offending group was higher than addiction onset offending group in “number of sexual offenses.” But both of “age of first offense” and “intervals of any offense” were significant higher in violence and property onset offending groups than the other onset groups. When we analyzed the victim type differences of different onset groups, the research found that sex onset offending group had more than 50% of adolescence type of victim, but in the addiction onset offending group was adult female type of victim.

#### Static-99

This scale makes assessments based on information in official documents and records from arrests of sexual offenders (such as demographic variables, related criminal records, and victims). The scale contains ten items, and a higher score represents a higher risk of recidivism (Helmus et al., 2012). Harris et al. (2003) conducted a study on sexual offenders in Canada and found that Static-99 is moderately accurate (ROC = 0.71) in predicting sexual recidivism. Studies in other countries including Sweden, the United Kingdom, and the United States reported an ROC ranging from 0.73 to 0.76 (Sjöstedt and Långström, 2001; Beech et al., 2002). Static-99 has been used in Taiwan. One of research team member, Dr. Huang, already personally contacted with one of author of Static-99 manual, Dr. Andrew Harris. We had his agreement of translating it into Chinese and used in some research reports (e.g., Chung and Hsu, 2008; Huang and Wu, 2012). Static-99 has been found good inter-rater reliability (kappa = 0.87 and 0.94) in Taiwan. All Static-99 was rated by trained therapist who followed the manual of Static-99 coding rules revised-2003 (Harris et al., 2003).

#### Dynamic Risk Factors

The dynamic risk factors are psychological markers that may related to recidivism. Those items also included the database system, they included two risk assessments: stable dynamic risk factors and acute dynamic risk factors. These scales were developed by Shen (2006) based on the above concept of etiological model of risk (Beech and Ward, 2004) and relevant researches of dynamic risk assessment (Hanson and Harris, 2000; Thornton, 2002). This risk scale could divide into four major constructs: sexual self-regulation, offense supportive cognitions, level of interpersonal functioning, and general self-regulation. All the risk assessment completed by trained therapist, they interviewed offender with index sexual offense and then follow manual compiled by Shen to rate each item. Dynamic risk items of scale could rate into three different certainty levels: 0 for no any signs of it, 1 for not so clear signs of it, and 2 for showing clear signs of it. Two risk assessments use the same certainty level to code, however, in the acute dynamic risk factors were according to any available information about sex offender within 1 month recently, but stable dynamic risk factors were retrospective for 3 to 6 months while assessment. In a later study, Shen indicated that the internal consistency coefficients of the two scales were, respectively, 0.73 and 0.82; the 2-month test-retest reliability of the stable dynamic risk factors ranged from 0.49 to 0.83, whereas the test-retest reliability of the acute dynamic risk factors ranged from 0.47 to 0.69. Both scales also had good inter-rater reliability (correlation ranging from 0.48 to 0.81) (Shen, 2006). We also included dynamic risk factors as one of the comparison variables of the different onset typologies. All the items of dynamic risk factors (acute and stable) refers to Appendix I.

#### Developmental Adversity

In this database system, they also included four items of developmental variables that related to risk of future re-offense, the trained therapist evaluated personal history of sexual offender throughout risk assessment. Items of developmental adversity included: Any childhood sexual victimization (under 12 years old), poor family relationship, maladaptive in school, and any conduct problems under 18. All items rated as yes (1) / no (0).

#### Criminal Specialization Index

To understand whether the criminal careers of sexual offenders converge or change, we referred to the method proposed by Harris (2012) to calculate criminal specialization and compared whether the criminal paths of different onset typologies led toward specialization or versatility. Based on the algorithm developed by Harris, two types of specialization indices can be derived for group comparison: the specialization threshold (ST), which is the number of crimes in a certain category committed by an offender divided by the total number of crimes committed, and specialization (with ST > 50% as the demarcation point), which, when the ST of a category of crimes is greater than 50%, indicates that the offender tends to focus on said type of crime.

### Data Analysis

Researchers interested in essential difference in onset typology of sexual offenders, therefore we use one-way ANOVA to do group comparison with offense-related variables, include prior any offense records, index offense information, sexual specialization, four areas of psychological markers, and developmental adversities.

## Results

Based on the criminal charge of the first offenses in the official criminal records, we divided the first offenses of the sexual offenders into four different onset typologies and then conducted one-way ANOVAs and chi-square tests using SPSS. The results are displayed in Table 2 through 5 and present the differences among the typologies in the various variables and the criminal specialization index.

The selected data in this study mainly originated from sexual offenses. As shown in Table 2, a sexual assault was the most common onset offense for the sexual offenders (nearly 60%), followed by property offenses (around 15%), violent offenses (around 10%), and finally, addiction.

### Group Comparison Regarding Criminal Specialization

The analysis of specialization in sexual offending in the various onset typologies in Table 3 shows that the number and proportion of those in the sexual onset offending group (i.e., those whose first onset offense was a sexual assault) who continued focusing on sexual offending later on were significantly higher than those in the other onset typologies. Specifically, over 20% of those in the sexual onset offending group focused most of their crimes on sexual offending; their ST was a high 0.92. In contrast, offenders whose onset offense was another type of crime were much less likely to focus on sexual offending; their ST values ranged from 0.28 to 0.36. Next, we analyzed the specialization on violent offenses among the various onset typologies and discovered that a violent crime as the onset offense exerted a substantial impact on the subsequent specialization. We further found that the average ST of violent crime in the violent onset offending group (i.e., those whose first onset offense was a violent assault) reached 0.5 and that nearly 20% had an ST greater than 50%. In comparison, the ST values of the other onset typologies were less than 0.05, thereby revealing that a violent crime as the onset offense has a significant impact on subsequent criminal behavior, which should not be underestimated. We then analyzed the specialization of property offenses and addiction, which led to similar results between the two. With property offenses or addiction, the type of the onset offense exerted a significant influence on the types of the subsequent offenses. ST values that are greater than 0.5 present these specialization tendencies, and 20 to 30% of each onset typology had ST values greater than 50%, which was statistically more than the proportions in the other onset typologies.

**TABLE 3:** Between onset-groups comparison with sexual specialization.

Type of Specialization Threshold	Sex	Violence	Property	Addiction	*χ^2^*(2)	*F* value
**For sexual crime**						
no. ST > 50% (%)	114 (23.4%)	3 (3.8%)	10 (8.3%)	0	547.98***	
ST scores	0.92 (0.18)	0.32 (0.20)	0.36 (0.19)	0.28 (0.20)		464.61***
**For violent crime**						
no. ST > 50% (%)	1 (0.2%)	15 (19.2%)	2 (1.7%)	0	159.20***	
ST scores	0.02 (0.09)	0.5 (0.21)	0.05 (0.12)	0.02 (0.07)		303.56***
**For property crime**						
no. ST > 50% (%)	2 (0.4%)	1 (1.3%)	27 (22.3%)	1 (2.7%)	171.97***	
STscores	0.03 (0.10)	0.07 (0.15)	0.53 (0.21)	0.05 (0.14)		352.68***
**For addiction**						
no. ST > 50% (%)	0	3 (3.8%)	0	12 (32.4%)	294.71***	
ST scores	0.01 (0.05)	0.05 (0.15)	0.02 (0.07)	0.59 (0.24)		397.74***

*The “specialization threshold” (ST) scores was proposed by Harris (2012), and is calculated by dividing the total number of charges for that type of offense (e.g., sexual) by the total number of charges for all offenses.*

*The 50% ST is commonly used to indicate the extent to an offender has a specialized criminal career.*

***p < 0.001.

### Group Comparison Regarding Psychological Markers

In the analysis above, we observed variations in the criminal careers of sexual offenders with different onset typologies. We further explored whether specialization and versatility can also reflect differences in four common psychological markers among sexual offenders. These psychological markers include the following: sexual self-regulation, offense supportive cognitions, the level of interpersonal functioning, and general self-regulation problems. Ward and Beech (2006) posited that personal vulnerabilities caused by certain growth and development experiences in sexual offenders are closely related to subsequent recidivism and indicated that corresponding variables can be identified using scales such as Static-99 and dynamic risk factors.

Using the data in Table 4, we can examine the differences among the onset typologies in psychological markers. In sexual self-regulation, significant group differences only existed for the victim being a stranger and for the sentencing date of prior convictions (the number of prior convictions ≥ 3). Further observations revealed that the sexual onset offending group was the lowest for both of these items; only about one out of every four offenders in the sexual onset offending group chose a stranger as their victim, and they also had a lower number of priors. This means that offenders in the sexual onset offending group tended to sexually assault those they knew, which also meant a greater chance of being exposed, arrested, and sentenced to prison. It is likely that the undesirable experience of being punished by the law also reduced the number of offenses that they committed. In terms of offense-supportive cognitions, we found that none of the items for this psychological marker reached the level of significance. This may indicate that no matter what the first offense was, once the offenders began sexual offending, something inside them compelled them to commit a sexual assault. In the level of interpersonal functioning, only poor social influence presented significant differences, with scores in the sexual onset offending group being much lower than those in the other three typologies. This could be due to the fact that this psychological marker item presents a uniqueness of sexual offending. For instance, most sexual offenders commit sexual assaults alone and regard them as personal secrets. Unlike other types of crime, sexual offenders are less likely to be goaded into offending, and this corresponds to the specialization phenomenon in the sexual onset offending group in Table 3. In general self-regulation problems, three variables in Static-99 reached the level of significance: non-sexual violence during sexual offense, prior conviction for non-sexual violence, and age under 25 years old. The first two are associated with the violent behavior of an offender, and the scores in the violent onset offending group were much higher than those in the other three typologies, the lowest of which occurred in the sexual onset offending group. The proportion of early onset was also the highest. In stable dynamic risk factors, scores for personal self-regulation traits in the sexual onset offending group were significantly lower than those in the other three typologies (a higher score indicates poorer self-regulation). In other words, those in the sexual onset offending group were not obviously impulsive because of fewer violent offense records.

**TABLE 4:** Onset-group comparison with four risk domains (psychological markers).

Risk domains	sex	violence	property	addiction	*χ^2^*(2)	*F* value
**Sexual self-regulation**						
**Static-99**						
Any convictions for non-contact sex offenses	0.02 (0.16)	0.01 (0.11)	0.01 (0.09)	0.03 (0.16)	2.02	
Unrelated victim	0.84 (0.37)	0.82 (0.39)	0.89 (0.31)	0.81 (0.40)	4.35	
Any Stranger victims	0.25 (0.43)	0.31 (0.47)	0.37 (0.49)	0.35 (0.48)	10.96*	
Any male victims	0.02 (0.16)	0.03 (0.16)	0.03 (0.18)	0.0 (0.00)	3.86	
Prior sex offenses	0.17 (0.43)	0.06 (0.25)	0.19 (0.43)	0.11 (0.31)		1.30
Prior sentencing dates	0.03 (0.18)	0.27 (0.45)	0.23 (0.42)	0.35 (0.48)	91.28***	
Stable dynamic risk scale						
Sexual self-regulation	0.45 (0.57)	0.46 (0.60)	0.55 (0.56)	0.3 (0.52)		1.39
**Pro-offending attitudes**						
**Stable dynamic risk scale**						
Attitude toward sexual violence	0.59 (0.63)	0.59 (0.65)	0.6 (0.61)	0.54 (0.69)		0.32
**Social-affective functioning**						
**Static-99**						
Single	0.66 (0.47)	0.59 (0.5)	0.65 (0.48)	0.57 (0.5)	7.75	
**Stable dynamic risk scale**						
Negative social influences	0.32 (0.53)	0.58 (0.75)	0.45 (0.62)	0.43 (0.65)		3.52**
Intimacy deficits	1.26 (0.79)	1.31 (0.83)	1.26 (0.77)	1.27 (0.80)		0.23
Social attachment problem	0.56 (0.61)	0.63 (0.58)	0.72 (0.66)	0.46 (0.56)		1.94
**Problem of general self-regulation**						
**Static-99**						
Index nonsexual violence	0.21 (0.41)	0.40 (0.49)	0.28 (0.45)	0.32 (0.48)	16.03**	
Prior nonsexual violence	0.09 (0.29)	0.55 (0.50)	0.26 (0.44)	0.27 (0.45)	107.96***	
Young	0.22 (0.41)	0.01 (0.11)	0.09 (0.29)	0.05 (0.23)	31.94***	
**Stable dynamic risk scale**						
Self-regulation tendency	0.93 (0.6)	1.03 (0.66)	1.22 (0.52)	1.11 (0.61)		5.84***
Cooperation with monitoring	0.09 (0.32)	0.13 (0.41)	0.18 (0.50)	0.11 (0.31)		1.41

* p < 0.05, **p < 0.01, *** p < 0.001.

### Differences Between Onset Typologies in Development Adversity

The theoretical models discussed by Marshall and Barbaree (1990); Ward and Beech (2006), and Smallbone and Cale (2015) regarding sexual offenders all mentioned that negative experiences during a sexual offender’s early growth and development stages influence their subsequent sexual criminal career. We therefore attempted to analyze and compare the developmental adversity variables of the different onset typologies in Table 5. We found significant differences in two items: poor relationships in family and experience of poor adaptation to school in the past. The property onset offending group had the highest proportion of offenders with the “poor relationships in family” item (approximately 35%), followed by the violent and sexual onset offending groups (approximately 24%), and lastly the addiction onset offending group. However, the “maladaptive in school” item presented a different trend: the violent onset offending group had the highest proportion of offenders (nearly 45%), followed by the property onset offending group.

**TABLE 5:** Onset-group comparison with experiences of developmental adversity.

Experiences of developmental adversity	Sex	Violence	Property	Addiction	*χ^2^*(2)
Any childhood sexual victimization	9 (1.8)	0	3 (2.5)	0	4.47
Poor family relationship	119 (24.4)	18 (23.1)	43 (35.5)	5 (13.5)	13.25*
Maladaptive in school	117 (24.0)	35 (44.9)	43 (35.5)	7 (18.9)	20.65***
Any conduct problems under 18	23 (4.7)	6 (7.7)	9 (7.4)	3 (8.1)	3.00

*p < 0.05, ***p < 0.001.

## Discussion

This study attempted to determine whether the onset typology of sexual offenders reflects differences in the nature of sexual offending using the perspectives of Thakker and Ward (2015) and Farrington (2003) regarding criminal careers. We utilized crime specialization and versatility, four risk factors, and growth and development experience to analyze the differences among sexual offenders with different onset typologies. The results confirmed that the onset typology of sexual offenders influences their criminal specialization, and some of the risk factors and growth and development experiences also varied with the onset typology. Some of our results support existing theories regarding sexual offending and are worth deliberating.

For the four risk factors, we only used Static-99 and a stable dynamic risk factor scale to analyze the differences among the sexual offenders with different onset typologies, and these methods were enough to elucidate some issues with proportional distribution. For instance, though weak, only one observed variable for offense-supportive cognitions could be analyzed, and consequently, the results could not show differences among the different onset typologies. This is a limitation of using samples from a database because the results can only be analyzed with a limited amount of information. Future studies involving psychological markers may improve on this by strengthening measured content. Those results of developmental adversity presented by the results above, it is worth considering that regarding the sexual onset offending group, the key influence was the onset offense and the certain degree of fixation that it created in the learning experience of the offender. For instance, the experience and feelings of committing a sexual assault influenced the evolution of future criminal behavior, and although only a little more than 20% of offenders specialized in sexual offending, their tendency to fixate on sexual offending is still a concern for society at large. In another aspect, the analysis above indicates that offenders with violent offenses, property offenses, and addiction presented the same specialization tendencies. From another perspective though, only 20 to 30% of these offenders will specialize in their respective offenses. In other words, no matter what the onset offense is, about two-thirds of offenders’ progress toward a versatile criminal career in the future. Thus, the results of this study indicate that to a certain degree, the onset typology of offenders will determine the development trajectories of their future criminal careers and leads them to specialize in a certain type of crime. However, criminal versatility is still an issue that requires attention. Based on these study results, it is worth deliberating how current correction and treatment policies can make arrangements for prison inmates who will return to society.

This study explored two criminal career characteristics: the onset typology and criminal specialization. Piquero et al. (2003) and Farrington (2003) compiled seven characteristics, such as desistance, career duration, and age of onset. Future studies can consider adding other criminal career characteristics to analyze the overall changes in the criminal trajectories of sexual offenders and identify the factors associated with desistance so as to obtain a more comprehensive understanding of the criminal careers of sexual offenders. Despite the many earlier theories regarding sexual offending, such as the integrated theory employed by Marshall and Barbaree (1990); Ward and Beech (2006), and Smallbone and Cale (2015) and the descriptive models used by Malamuth (2003) and Hudson and Ward, the research results and clinical experiences collected by researchers all indicate that significant events during the growth and development of sexual offenders influence their subsequent life trajectories and gradually lead them toward sexual offending. However, most existing studies have focused on the development experiences, background factors, or distal factors on parental care and abuse experiences during childhood and have rarely paid attention to the influence of criminal onset. Even if the influence of the criminal onset was discussed, most discussions focused on the correlation between age of onset and recidivism.

Those results of developmental adversity support the views of Marshall and Barbaree (1990) and Smallbone and Cale (2015) regarding the integrated theory of sexual offending, in that problems with early attachment can gradually evolve into poor social control and are associated with the risk of sexual offending. We found that a sizeable proportion of sexual offenders had problems with their familial relationships and adaptation to school; thus, when they later escaped the constraints of social control, the likelihood of them committing a crime increased. One matter worthy of discussion is the sexually assaulted during childhood item. The proportion of offenders with this experience was low in every group. There have been theories and notions of the effects of sexual abuse in childhood in previous studies, indicating that those who were victims of sexual assaults as a child grow up to be offenders themselves, which is included in the etiological model proposed by Beech and Ward (2004). However, our analyses found that other development trajectories might require attention instead because the proportions of those are higher than those who were victimized as children. Moreover, what stimulates the onset of sexual offending may not necessarily be associated with being sexually assaulted as a child; for instance, experiences of improper physical and mental development such as attachment issues, physical abuse, or lack of care may all give rise to psychological vulnerability that gradually puts an offender on the path to a sexual criminal career.

Ward and Beech (2006) and Smallbone and Cale (2015) emphasized the mutual influence between the biological and environmental factors of offenders and their criminal behavior. In other words, the criminal onset of a sexual offender is not merely a marker of a criminal record (prior conviction); it may also reappear and be influenced by biological and environmental factors, thereby contributing to the next offense. The analysis of crime specialization and versatility in this study revealed another matter worth deliberating: that the population of sexual offenders consists of sexual offenders with various characteristics. Only by understanding these essential differences can the right therapy plans be made to help reduce future rates of sexual recidivism.

## Data Availability Statement

The raw data supporting the conclusions of this article will be made available by the authors, without undue reservation.

## Ethics Statement

The studies involving human participants were reviewed and approved by the Institutional Review Board of Fu Jen Catholic University (project approval no. C103152). Written informed consent to participate in this study was provided by the participants’ legal guardian/next of kin.

## Author Contributions

All authors listed have made a substantial, direct, and intellectual contribution to the work, and approved it for publication.

## Conflict of Interest

The authors declare that the research was conducted in the absence of any commercial or financial relationships that could be construed as a potential conflict of interest.

## Publisher’s Note

All claims expressed in this article are solely those of the authors and do not necessarily represent those of their affiliated organizations, or those of the publisher, the editors and the reviewers. Any product that may be evaluated in this article, or claim that may be made by its manufacturer, is not guaranteed or endorsed by the publisher.

## Appendix I

Stable Dynamic Risk Factors

(coding rules: 0 = no any signs, 1 = not so clear signs, and 2 = show clear signs)

1. Any negative social influence

2. Any intimacy deficits

3. Any social attachment deficits

4. Any problem of sexual self-regulation

5. Any pro-offending attitudes about sex

6. Non-compliance to monitoring

7. Any problem of self-management

→sum of item1 to item7 as a stable risk potential.

Acute Dynamic Risk Factors

(coding rules: 0 = no any signs, 1 = not so clear signs, and 2 = show clear signs)

1. Any opportunity of victim access

2. Emotion dysregulation

3. disrupt social support or network

4. Hostility to others

5. Alcohol or drug abuse

6. Sexual preoccupation

7. reject monitoring

8. Any other situations related to re-offense

→sum of item1 to item8 as an acute risk level.
